# Synergistic effect of westerly circulation and western North Pacific anticyclone on interannual variation of mid-spring rainfall in southeastern China

**DOI:** 10.1371/journal.pone.0323244

**Published:** 2025-05-15

**Authors:** Wenxu Lu, Xiaodong Liu

**Affiliations:** 1 State Key Laboratory of Loess Science, Institute of Earth Environment, Chinese Academy of Sciences, Xi’an, China; 2 University of Chinese Academy of Sciences, Beijing, China; Balochistan University of Information Technology Engineering and Management Sciences, PAKISTAN

## Abstract

The spring rainfall in southeastern China (SEC) is a unique climatic phenomenon and has important impacts on local socio-economics. In this study, the climatological characteristics of the SEC mid-spring (mid-March to early May) rainfall and the causes of its interannual variations are systematically analyzed using precipitation observations from 1961 to 2022. The results show that the interannual variations of SEC rainfall are mainly influenced by the westerly circulation (WC) south of the Tibetan Plateau, the western North Pacific anomalous anticyclone (WNPAC), and their synergistic effects. The WC is modulated by the mid-latitude Eurasian atmospheric wave train (AWT) and the thermal effects of the Tibetan Plateau. When the AWT is in a positive (negative) phase or the Tibetan Plateau experiences a cold (warm) spring, the WC and the bypassing flow on the plateau’s southeastern side strengthen (weaken), leading to excessive (deficient) SEC rainfall. During El Niño (La Niña) events in winter or spring, the warmer (cooler) tropical eastern Pacific forces Rossby waves to propagate westward, triggering an anomalous anticyclone (cyclone) in the northwest Pacific. This intensifies (weakens) the southwesterly flow on its western flank, ultimately enhancing (suppressing) SEC rainfall. More importantly, when these factors simultaneously exhibit simultaneous anomalous signals, the synergistic effect of the WC and WNPAC may become a key factor in forming extreme events of mid-spring rainfall in the SEC. This study helps deepen our understanding of the regional rainfall variation mechanisms and has the potential to be applied to short-term climate prediction.

## 1. Introduction

**Fig 1:**
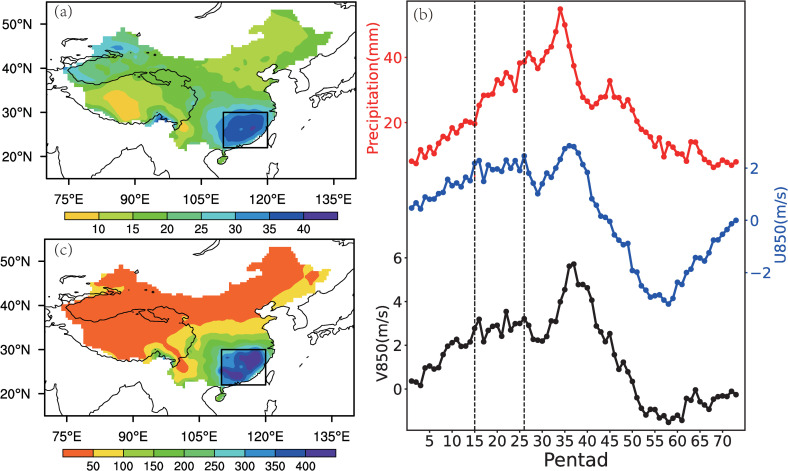
(a) Spring (March-May) precipitation as a percentage of annual total in China (1961–2022). **(b) Annual cycles of 1961–2022 average pentad precipitation (mm), 850-hPa zonal and meridional wind speeds (m/s) over the SEC region. (c) Mid-spring (15^th^-26^th^ pentad) average precipitation (mm) in China (1961–2022).** The black box in (a) and (c) marks the SEC region (22°-30°N, 110°-120°E), with the black contour indicating the 3000m Tibetan Plateau topography. Vertical dashed lines in (b) indicate the mid-spring period (15^th^-26^th^ pentad).

Spring rainfall in southeastern China (SEC) is a unique climatic phenomenon distinct from monsoon precipitation in the East Asian monsoon region [1]. It accounts for over 35% of the region’s annual precipitation (Fig 1a). Due to its high interannual variability, extreme precipitation or drought events in spring pose significant challenges to the social development of this densely populated region, particularly affecting agricultural production during growing seasons [2–4]. Therefore, the characteristics and physical mechanisms of the SEC spring rainfall have long been a focus of research.

The mechanisms of the SEC spring rainfall have been extensively studied, revealing that this phenomenon is influenced by a variety of forcings and associated atmospheric circulation anomalies. The spring rainfall in the SEC is typically supplied by two main water vapor pathways, the westerly circulation south of the Tibetan Plateau and the southwesterly flow in the western North Pacific [5]. The El Niño-Southern Oscillation (ENSO), closely associated with sea surface temperature (SST) anomalies in the tropical Pacific, is a major driver of interannual variability in the SEC spring rainfall [6–9]. For example, a strong El Niño event triggers a teleconnection between the Central Pacific and East Asia, characterized by anomalous anticyclonic circulation in the lower troposphere over the northwestern Pacific. This teleconnection can persist from winter to the following spring or early summer [10,11], enhancing the southwesterly flow as a key water vapor channel. Feng and Li [2] further distinguished the impacts of different ENSO types, demonstrating El Niño Modoki events may lead to negative spring precipitation anomalies compared to typical El Niño events. The synergistic impact of the Pacific Decadal Oscillation and ENSO may also modulate the inter-decadal variability in SEC spring rainfall [12,13].

Indian Ocean SST anomalies also play a key role in East Asian spring rainfall. The Indian Ocean subtropical dipole signal, originating in the previous winter, modulates East Asian precipitation through anomalous Hadley circulation [14,15]. Phase differences exist between the impacts of Pacific and Indian Ocean SST anomalies on spring rainfall in southern China [16]. Additionally, the interannual variability of spring rainfall is jointly modulated by the North Atlantic Oscillation and the North Pacific Oscillation [17]. Furthermore, winter Arctic sea ice reduction can enhance Eurasian wave trains, strengthening the subtropical westerly flow and promoting East Asian spring precipitation [18].

In addition to SST effects, anomalies in land thermal conditions also significantly influence SEC spring rainfall. For example, Eurasian snowpack, particularly over the western Tibetan Plateau in the previous autumn, can induce East Asian precipitation anomalies by altering the strength and location of the westerly jet [19,20]. In recent decades, global warming has led to a reduction in Eurasian snowpack, potentially weaken the westerly wind circulation and reduce SEC rainfall in early spring [21]. The thermal effects of the Tibetan Plateau also significantly impact East Asia’s spring climate. Numerical experiments demonstrate that sensible heating over the southeastern Tibetan Plateau from March to early May enhances southwesterly flow on its southeastern flank, thereby promoting SEC spring rainfall [22,23].

While many studies have examined SEC spring rainfall, most have focused on individual factors in isolation. This paper systematically analyzes the climatological characteristics and interannual variability of SEC spring rainfall using the latest precipitation observations from China over the past 62 years (1961–2022). This study aims to deepen our understanding of the physical mechanisms underlying SEC spring rainfall and to improve short-term climate predictions in the region. Special attention is given to the synergistic effects of the westerly circulation south of the Tibetan Plateau and the western North Pacific anomalous anticyclone, both of which are modulated by land and ocean thermal conditions. The rest of the paper is organized as follows: Section 2 describes the data and methods used. Section 3 assesses the climatological characteristics of spring precipitation in East Asia and the main influencing factors, while also analyzing the physical mechanisms and synergistic interactions contributing to the SEC spring rainfall. Finally, Section 4 provides a summary and discussion.

## 2. Data and methods

The daily gridded precipitation data used are produced by the National Climate Center of the China Meteorological Administration [24], obtained as the interpolated result by using the thin-plate smoothing spline technology based on observations from over 2400 national stations (including basic, baseline, and general stations) in China. This dataset ranges across all of China from 1961 to 2022, with both high spatial resolution of 0.25° and daily-scale temporal resolution, and is highly recognized and widely used [25–27]. Due to the large variations of daily precipitation on a climatic scale, it has been recalculated as pentad mean precipitation data in this study.

To analyze the atmospheric circulation characteristics in spring, the ERA5 reanalysis dataset from the European Centre for Medium-Range Weather Forecasts (ECMWF) is used. This dataset covers the period from 1940 to the present, with 31-km grid resolution and 137 vertical levels, including atmospheric, land, and ocean variables [28]. The selected meteorological variables include winds, geopotential, air temperature, surface sensible heat flux, surface latent heat flux, and surface net longwave radiation, which are aligned temporally with the precipitation data.

SST data are obtained from the Hadley Centre’s reconstructed sea ice and sea surface temperature dataset (HadISST1), with a spatial resolution of 1° × 1°. This dataset is based on ship and satellite observations and has been optimally statistically processed [29]. To compare the effects of SST anomalies in different regions, the ENSO indices defined by regionally averaged SST as summarized by Capotondi and Sardeshmukh [30] are calculated as shown in Eq. 1:


$$\left\{ \begin{array}{c} {Nino}_{1+2}=\overset{―}{SST}(0-10{}^{\circ}S,90{}^{\circ}W-80{}^{\circ}W)\\ {Nino}_{3}=\overset{―}{SST}(5{}^{\circ}N-5{}^{\circ}S,\;150{}^{\circ}W-90{}^{\circ}W)\\ {Nino}_{3.4}=\overset{―}{SST}(5{}^{\circ}N-5{}^{\circ}S,\;170{}^{\circ}W-120{}^{\circ}W)\\ {Nino}_{4}=\overset{―}{SST}(5{}^{\circ}N-5{}^{\circ}S,\;160{}^{\circ}E-150{}^{\circ}W) \end{array}\right.\;$$ (1)

This study employs several commonly used statistical analysis methods, including correlation analysis, multiple regression analysis, empirical orthogonal function (EOF) decomposition, and composite analysis. Additionally, the wave activity flux $\vec{w}$ (Eq. 2) is calculated to characterize Rossby wave propagation [31]:


$$\vec{w}=\;\frac{p\cos\varphi }{2\left|\vec{V}\right|}\left\{ \begin{array}{c} \frac{U}{a^{2}\cos^{2}\varphi }\left[{\left(\frac{\partial \psi^{\prime }}{\partial \lambda }\right)}^{2}-\psi^{\prime }\frac{\partial^{2}\psi^{\prime }}{\partial \lambda^{2}}\right]+\frac{V}{a^{2}\cos^{2}\varphi }\left[\frac{\partial \psi^{\prime }}{\partial \lambda }\frac{\partial \psi^{\prime }}{\partial \varphi }-\psi^{\prime }\frac{\partial^{2}\psi^{\prime }}{\partial \lambda \partial \varphi }\right]\\ \frac{U}{a^{2}\cos^{2}\varphi }\left[\frac{\partial \psi^{\prime }}{\partial \lambda }\frac{\partial \psi^{\prime }}{\partial \varphi }-\psi^{\prime }\frac{\partial^{2}\psi^{\prime }}{\partial \lambda \partial \varphi }\right]+\frac{V}{a^{2}}\left[{\left(\frac{\partial \psi^{\prime }}{\partial \varphi }\right)}^{2}-\psi^{\prime }\frac{\partial^{2}\psi^{\prime }}{\partial \varphi^{2}}\right] \end{array}\right.\;$$ (2)

where $\left|\vec{V}\right|$ represents the intensity of the horizontal wind, φ and λ denote latitude and longitude, respectively, p is the ratio of pressure to standard pressure (1000 hPa), U and V are respectively the zonal and meridional wind speeds, a is the Earth’s radius, and $\psi^{\prime }$ represents the perturbation of geopotential.

To reflect the thermal effect of the Tibetan Plateau, the intensity of the surface heat source of the Tibetan Plateau (abbreviated as SHS, Eq. 3) is assessed according to the definition by Ye and Gao [32]:


SHS=SH+LH+LR (3)

where SH, LH, LR represent the surface sensible heat flux, latent heat flux and net longwave radiation, respectively. Additionally, the standardized SHS series averaged over the plateau area above 3000 m is defined as the SHS Index (SHSI) of the Tibetan Plateau.

Referring to Rasmusson [33], we calculate the whole-layer water vapor budget (WVB) (Eq. 4):


$$WVB=\int_{p_{u}}^{p_{s}}WVT\;dp$$ (4)

where WVT represent the total water vapor transport at a pressure layer (Eq. 5) and is calculated through the moisture transport across the four boundaries (Eq. 6):


$$WVT\;={WVT}_{W}+{WVT}_{S}-{WVT}_{E}-{WVT}_{N}$$ (5)

$$\left\{ \begin{array}{c} {WVT}_{W}=\;\frac{1}{g}\int_{\varphi_{1}}^{\varphi_{2}}q_{v}u\left(\lambda_{1},p\right)d\varphi \\ {WVT}_{E}=\;\frac{1}{g}\int_{\varphi_{1}}^{\varphi_{2}}q_{v}u\left(\lambda_{2},p\right)d\varphi \\ {WVT}_{S}=\;\frac{1}{g}\int_{\lambda_{1}}^{\lambda_{2}}q_{v}v\left(\varphi_{1},p\right)d\lambda \\ {WVT}_{N}=\;\frac{1}{g}\int_{\lambda_{1}}^{\lambda_{2}}q_{v}v\left(\varphi_{2},p\right)d\lambda \end{array}\right.\;$$ (6)

where $q_{v}$ is specific humidity, u zonal wind, v meridional wind, φ latitude, λ longitude, $p_{s}$ the surface pressure, $p_{u}$ the pressure of top layer (200 hPa), and g the acceleration of gravity. ${WVT}_{W}$, ${WVT}_{E}$, ${WVT}_{S}$, and ${WVT}_{N}$ denote the horizontal water vapor transport (flux) across each side of a budget area (i.e., west, east, south, and north, respectively) at a pressure layer.

## 3. Results

### 3.1. Precipitation climatology and related circulations

We first analyze the climatological characteristics of spring rainfall in China. According to the distribution of the ratio of precipitation in spring (March-May) to the annual total over mainland China during 1961–2022 (Fig 1a), the highest spring rainfall occurs in the SEC (22°-30°N, 110°-120°E). Fig 1b illustrates the climatological (1961–2022) annual cycles of pentad precipitation, 850-hPa meridional and zonal wind speeds averaged over the SEC. From winter to early summer (the 34^th^ pentad), precipitation in the SEC increases continuously (Fig 1b), while changes in wind speeds show less consistency. Although zonal and meridional winds in the lower troposphere over the SEC generally strengthen during the transition period from winter to summer, the wind increase slows significantly and peaks during the 15^th^–26^th^ pentads (mid-March to early May), corresponding to the spring rainy season in the SEC. Subsequently, the zonal and meridional winds experience a brief weakening, followed by a second significant increase from approximately the 31^st^ pentad (early June) to the 36^th^ pentad (late June) (Fig 1b), coinciding with the full onset of the East Asian summer monsoon. During the monsoon, the primary rainband in East Asia shifts northward, forming the ‘Meiyu’ rainfall over the Yangtze-Huaihe River basin [34,35]. Therefore, it is evident that spring rainfall and summer monsoon rainfall in the SEC represent two distinct phases. The period from the 15^th^ to the 26^th^ pentad, corresponding to the first peak phase of zonal and meridional winds, is defined as the mid-spring period in this study, consistent with previous studies [22]. According to the distribution of precipitation during the mid-spring period (Fig 1c), the SEC region generally receives more than 250 mm of rainfall, with some areas exceeding 400 mm, making it the most significant spring rainfall region in China. For the convenience of statistical analysis in the following sections, the standardized mid-spring rainfall series over the SEC is defined as the Rainfall Index (RI, Eq. 7):


$$RI=std(\overset{―}{\mathrm{Pr}}(22{}^{\circ}-30{}^{\circ}N,110{}^{\circ}-120{}^{\circ}E))$$ (7)

where $\overset{―}{\mathrm{Pr}}$ represents the regional average of precipitation, and std represents the standardization process.

**Fig 2:**
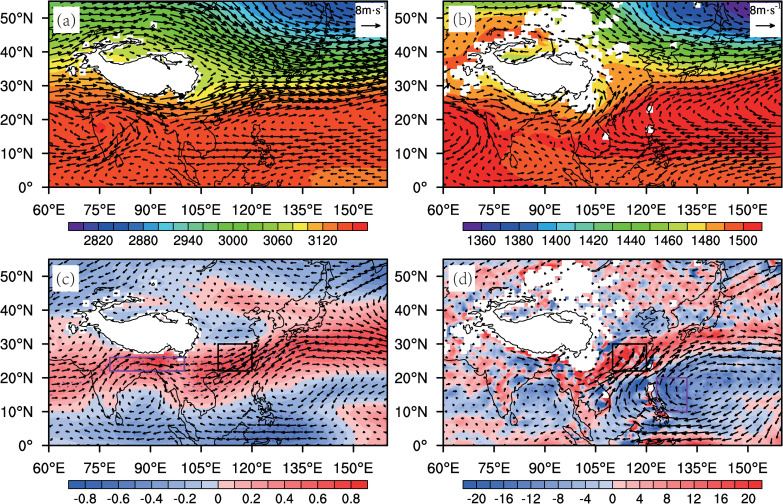
(a, b) 1961–2022 mid-spring average horizontal wind (vector, m/s) and geopotential height (shaded, gpm) fields at (a) 700 hPa and (b) 850 hPa. **(c) Mid-spring 700-hPa wind (vector, m/s) and zonal wind speed (shaded, m/s) regressed against concurrent RI (1961–2022). (d) As (c) but for 850-hPa wind (vector, m/s) and relative vorticity $(shaded,\;s^{-1}$).** Black boxes in (c) and (d) mark the SEC region; Purple boxes denote key regions for the Tibetan Plateau south side (c) and tropical western Pacific (d). Dotted areas indicate significance at the 99% confidence level.


Spring marks the transition of East Asian atmospheric circulation from winter to summer. During this period, the upper-tropospheric mid-latitude westerly jet rapidly weakens and shifts northward [36,37]. However, on average, the westerly circulation south of the Tibetan Plateau still dominates in the mid-troposphere (700 hPa). Due to the blocking effect of the plateau’s topography, the westerly flow is deflected around the eastern side of the plateau, thereby influencing the climate of the SEC region (Fig 2a). Meanwhile, in the lower troposphere (850 hPa), the southwesterly airflow on the western side of the western Pacific subtropical high transports a large amount of moisture to the SEC (Fig 2b), which also reveals the two main moisture transport pathways for the SEC spring rainfall [5]. Regression of the wind fields onto the RI further confirms these dominant circulation systems (Fig 2c, d): the westerly circulation south of the plateau (hereafter abbreviated as WC) and the western North Pacific anomalous anticyclone (WNPAC). Various external forcing factors, such as Pacific, Indian, and Atlantic SST anomalies, as well as Eurasian land thermal forcing, typically affect the strength of these two circulations [38,39], and ultimately impact SEC spring rainfall. Therefore, this study focuses on WC and WNPAC as key dynamical frameworks. To facilitate the quantitative description of the interannual variations of WC and WNPAC, the 700-hPa WC index (WCI, Eq. 8) and the WNPAC index (WNPACI, Eq. 9) are defined based on the regionally averaged 700-hPa zonal wind speed in the key area (Fig 2c, brown box) and the 850-hPa relative vorticity averaged for the significant regression area (Fig 2d, brown box), respectively:

$$WCI=std(\overset{―}{U_{700}}(22{}^{\circ}-26{}^{\circ}N,78{}^{\circ}-100{}^{\circ}E))$$ (8)

$$WNPACI=-std(\overset{―}{{Vort}_{850}}(10{}^{\circ}-20{}^{\circ}N,123{}^{\circ}-132{}^{\circ}E))$$ (9)

where $\overset{―}{U_{700}}$ represents the regionally averaged 700-hPa zonal wind, and $\overset{―}{{Vort}_{850}}$ represents the regionally averaged 850-hPa vorticity.

**Fig 3:**
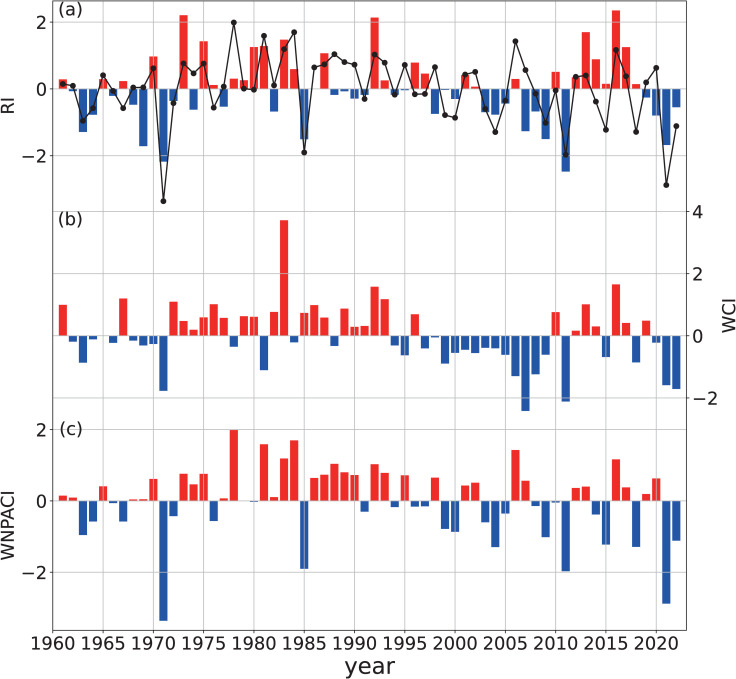
1961-2022 time series of standardized indices: (a) RI, (b) WCI, and (c) WNPACI. The black line (a) shows the linear regression of RI $\left({RI}_{reg}\right)$ using WCI and WNPACI.


Fig 3 shows that the interannual variations of the RI are highly consistent with those of the WCI and the WNPACI. The regression results of the WCI, WNPACI, or their combination onto the RI indicate that all of them have significant positive correlations with the RI (Table 1). This implies that the enhancement of the upstream WC or downstream WNPAC in spring is conducive to an increase in the SEC spring rainfall. The regression equation of the combined WCI and the WNPACI onto the RI is shown in Eq. 10:

**Table 1:** Correlation coefficients and explained variances between the mid-spring Rainfall Index (RI) and circulation indices (1961–2022).

	correlation coefficient	explained variance
WCI	$0.58^{***}$	33%
WNPACI	$0.63^{***}$	39%
WCI+ WNPACI	$0.72^{***}$	52%

(^***^ denotes significance at the 99% confidence level).

$${RI}_{reg}=\;0.39WCI+0.47WNPACI$$ (10)

The correlation coefficient between ${RI}_{reg}$ and the observed RI is 0.72 (explained variance: 52%), which is significantly better than the regression results of a single factor (Table 1). It indicates that for the interannual variation of the SEC spring rainfall, the synergistic effect of the WCI and WNPAC is more important. It should be noted that the WCI and WNPACI have a certain positive correlation, with a correlation coefficient of 0.38 (r = 0.38), which means that there may be an interaction between them. However, partial correlation analyses show that the partial correlation coefficient of the WCI (WNPACI) with the RI is 0.46 (0.53) after removing the mutual influence of WCI and WNPACI. This indicates that although these two factors are not completely independent, they can still independently affect SEC rainfall. Besides, WNPAC may play a greater role in the SEC spring rainfall. In the following sections, we further analyze the potential physical mechanisms influencing the variations of these two factors in detail.

### 3.2. Mechanisms of WC variations

**Fig 4:**
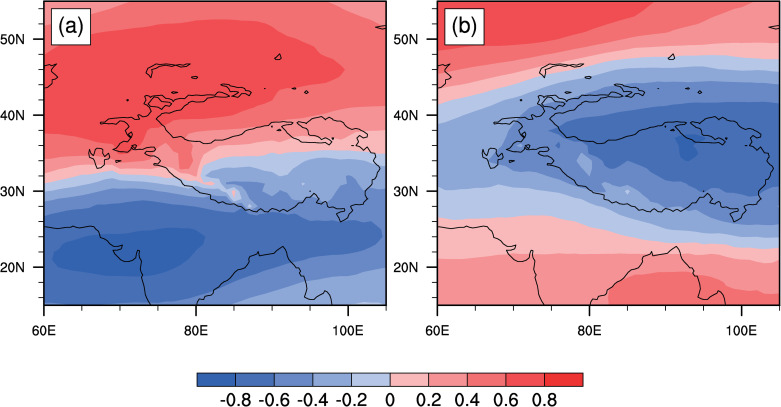
EOF modes of 1961-2022 500-hPa zonal wind: (a) first mode, (b) second mode.

**Fig 5:**
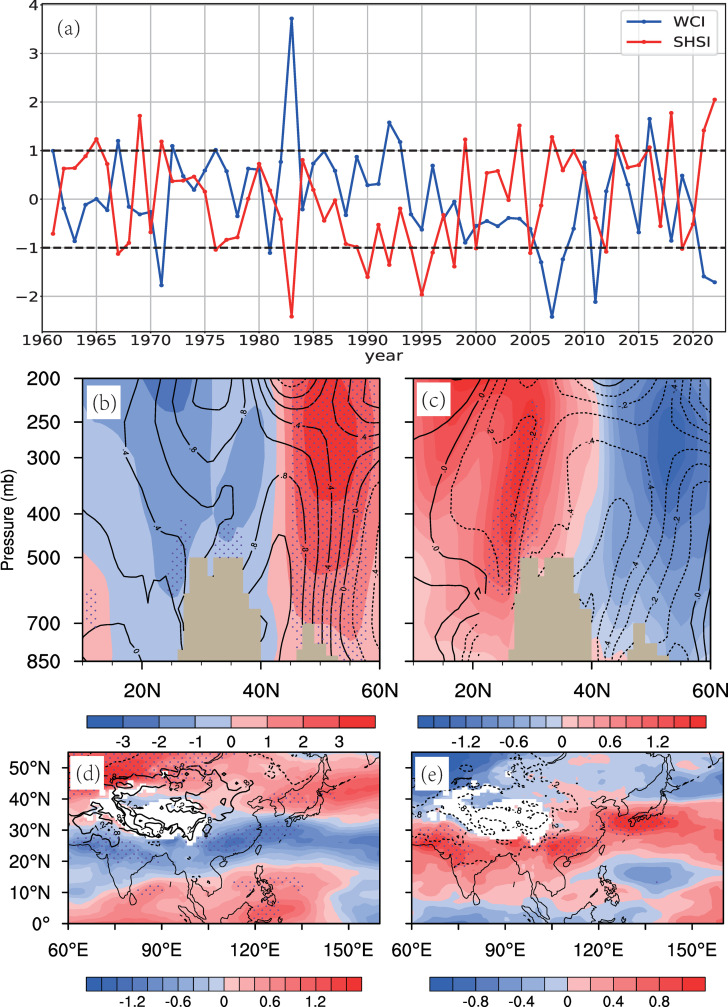
(a) 1961–2022 standardized mid-spring SHSI (red) and WCI (blue), with dashed lines indicating ±1 standard deviation. **(b, c) Latitude-pressure cross sections (78°-100°E average) of zonal wind (shaded, m/s) and are temperature (contours, K) anomalies for (b) strong and (c) weak SHSI years. (d, e) 700-hPa zonal wind (shaded, m/s) and surface air temperature (contours, K) anomalies for (d) strong and (e) weak SHSI years.** Anomalies in (b)-(e) are relative to 1961–2022. Brown/white areas mark the Tibetan Plateau topography; dotted areas indicate 90% significance.

**Fig 6:**
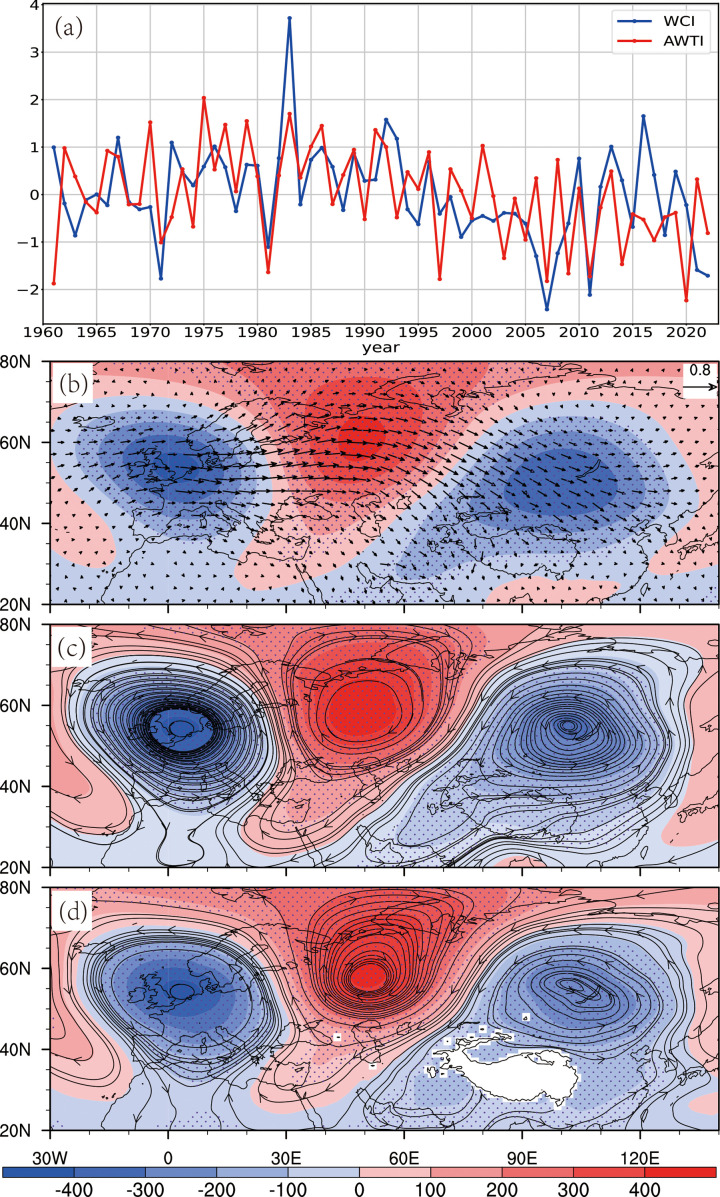
(a) Year-to-year variations of standardized mid-spring AWTI (red) and WCI (blue), 1961–2022. **(b) 200-hPa wave activity flux (vector, m² ∙ s ⁻ ²) and geopotential height (shading, gpm) regressed onto mid-spring AWTI. (c) 500-hPa wind (streamline, m/s) and geopotential height (shading, gpm) regressed onto mid-spring AWTI. (d) As (c) but for 700-hPa**. Dotted areas indicate 90% significance.

Climatologically, the tropospheric westerly circulation predominantly controls the southern Tibetan Plateau in spring (Fig 2a), serving as a key driver of SEC spring rainfall. The changes in westerly winds are influenced by many factors. Prior research indicates that the plateau’s westerly circulation responds to thermal forcing from the Tibetan Plateau surface heat source (SHS) [40] and Eurasian atmospheric wave trains (AWT) [38]. To identify the most significant influencing factors, we conducted an EOF decomposition of the 500 hPa zonal wind field. The results show that the first two modes explain 38.6% and 18.5% of the variance, respectively (Fig 4). The first EOF mode captures the thermal forcing signal, demonstrating a significant correlation (r = 0.65, P < 0.01) between the PC1 time series and the plateau SHS Index (SHSI) (Fig 5a). This mode shows a dipole pattern in the westerly winds around the plateau, characterized by a north-south reversal (Fig 4a). The second mode is associated with wave train signals in the upper troposphere, with a correlation coefficient of -0.52 (P < 0.01) between the PC2 and the AWT Index (AWTI) (Fig 6a). This mode exhibits a consistent spatial pattern in the westerly winds around the plateau (Fig 4b). These findings indicate that thermal forcing at the surface of the plateau and wave train variations in the upper layers are the primary factors driving changes in westerly circulation around the plateau.

The combined effect of SHSI and AWTI explains 30% of the WCI variance (r = 0.55), highlighting their joint modulation of WC variability (Table 2). This further confirms that the spring WC is indeed modulated by the SHS and AWT. Additionally, there is a weak correlation between the SHSI and AWTI, with a correlation coefficient of -0.21, which may be linked to the influence of AWT on Eurasian temperatures [41]. However, the triggering mechanism of the AWT in spring is complex and falls outside the scope of this study. In the next section, the physical processes through which the SHS and AWT affect the WC are discussed in detail .

**Table 2:** Correlation coefficients and explained variances between the mid-spring Westerly Circulation Index (WCI) and influencing factors (1961-2022).

	correlation coefficient	explained variance
SHSI	$0.44^{***}$	19%
AWTI	$0.41^{***}$	17%
SHSI + AWTI	$0.55^{***}$	30%

(^***^ denotes significance at the 99% confidence level).

#### 3.2.1. Thermal effects of the Tibetan Plateau.

Due to its high altitude, the surface heating of the Tibetan Plateau can directly affect the mid-troposphere, thereby influencing the surrounding and even global climates [42–44]. For example, previous studies have demonstrated that SEC spring rainfall was closely related to sensible heat anomalies in the southeastern part of the Tibetan Plateau [22,23]. Additionally, evidence suggests that the thermal effects of the Tibetan Plateau can influence the downstream climate by modulating the intensity and position of the subtropical westerly jet [45–47].

Building on the aforementioned close relationship between the WC and SEC spring rainfall, this study further emphasizes the role of the plateau’s thermal forcing on the westerly circulation. Using one standard deviation of the spring SHSI series as a threshold (Fig 5a), we identified 11 warm years (1965, 1969, 1971, 1999, 2004, 2007, 2013, 2016, 2018, 2021, 2022) and 12 cold years (1967, 1976, 1983, 1990, 1992, 1995, 1996, 1998, 2000, 2005, 2012, 2019) for the Tibetan Plateau. The corresponding composite analyses indicate that zonal wind anomalies on the north and south sides of the Tibetan Plateau exhibit a dipole pattern during warm and cold years (Fig 5b–e). In warm (cold) years, the westerlies at 700 hPa south of the plateau weaken (strengthen), while those north of the plateau strengthen (weaken) (Fig 5d, e). This pattern of opposite changes in the westerly circulations on the north and south sides of the plateau aligns with variations in the meridional temperature gradient (Fig 5b, c). These results show that the thermal effects of the plateau in spring significantly influence the position and strength of the westerly circulation, which is consistent with findings from previous numerical experiments [46].

#### 3.2.2. Atmospheric wave train.

The springtime atmospheric wave train (AWT) over the North Atlantic to East Asia is a zonal teleconnection that describes the dominant pattern of westerly wind fluctuations [38]. The AWT derives its energy from the westerly flow and is generally considered to result from the zonal propagation of Rossby waves [38]. Additionally, it can significantly influence the climate in Eurasia [41,48].

To explore the relationship between the AWT and WC, this study adopts the definition of the AWTI by Chen et al. [38], which represents the principal component series of the first EOF mode of the meridional wind at 250 hPa over the Eurasian region (35°N-75°N, 80°W-120°E). Statistical analysis shows that the correlation coefficient between the AWTI and WCI is 0.38 (p < 0.01) (Fig 6a). Regression analysis of wave activity flux at 200 hPa against the AWTI reveals the basic characteristics of Rossby wave zonal propagation, with the wave source mainly located in Western Europe and propagating eastward along the mid-latitude westerly belt (40°N-70°N) (Fig 6b). The geopotential height and wind fields at different tropospheric levels regressed against the AWTI (Fig 6b–d) indicate that when the AWT is in a positive (negative) phase, the geopotential height in the mid-to-upper troposphere over the Eurasian continent exhibits a consistent “-, +, -” (“+, -, +”) anomaly pattern, accompanied by the corresponding anomalous circulations. Under the control of an anomalous cyclone (anticyclone) over the Tibetan Plateau, the westerly circulation south of the plateau is enhanced (weakened) (Fig 6b–d).

### 3.3. Mechanisms of WNPAC variations

Another major circulation factor affecting the SEC spring rainfall is the WNPAC (Fig 2d). This anomalous lower-tropospheric anticyclone is closely linked to oceanic thermal forcing [49]. On interannual timescales, the WNPAC is associated with El Niño events, while on decadal timescales, the Pacific Decadal Oscillation may modulate its variability [50]. Typically, after the mature phase of an El Niño event in winter, the WNPAC develops and persists until the following summer. Studies suggest that SST anomalies in the tropical Atlantic and North Indian Ocean can also promote WNPAC formation by generating Rossby and Kelvin waves, respectively [51,52]. The WNPAC enhances southwesterly airflow and moisture transport toward the SEC, thereby increasing precipitation over southern China [39,53].

Given the strong WNPAC-ENSO relationship, interannual correlation coefficients are calculated between the spring (March-May) WNPACI and the preceding/concurrent monthly Niño indices (Table 3). The highest correlation coefficient (r > 0.46, p < 0.01) occurs between the ${February\;Nino}_{3}$ index and the spring WNPACI, indicating that eastern Pacific SST anomalies are more closely tied to the anomalous anticyclone affecting SEC rainfall. This one- to two-month lagged correlation implies that the preceding SST anomalies may offer a predictive signal for the SEC spring rainfall. Thus, the ${Nino}_{3}$ index is used hereafter to represent ENSO strength.

**Table 3:** Correlation coefficients between spring WNPACI and monthly Nino indices (November- April, preceding–concurrent year).

	${Nov.}^{-1}$	${Dec.}^{-1}$	Jan.	Feb.	Mar.	Apr.
${Nino}_{1+2}$	$0.32^{**}$	$0.31^{**}$	$0.34^{***}$	$0.4^{***}$	$0.29^{**}$	$0.21^{*}$
${Nino}_{3}$	$0.35^{***}$	$0.37^{***}$	$0.38^{***}$	$0.46^{***}$	$0.45^{***}$	$0.31^{**}$
${Nino}_{3.4}$	$0.35^{***}$	$0.39^{***}$	$0.39^{***}$	$0.42^{***}$	$0.43^{***}$	$0.34^{***}$
${Nino}_{4}$	$0.28^{**}$	$0.34^{***}$	$0.33^{***}$	$0.31^{**}$	$0.29^{**}$	0.15

(^*^, ^**^, ^***^ denote significance at the 90%, 95%, 99% confidence levels, respectively. The bold numbers indicate correlation coefficients > 0.4, and the superscript -1 denotes the preceding year.).

**Fig 7:**
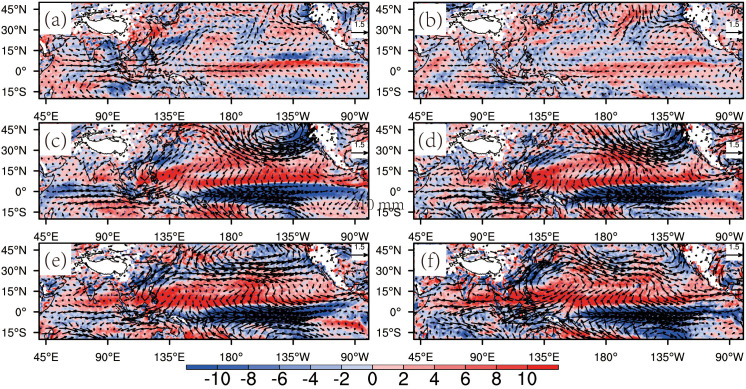
Concurrent 850-hPa wind (vector, m/s) and vertical velocity (shading, pa/s) regressed onto the Nino3 index in (a) November, (b) December, (c) January, (d) February, (e) March, and (f) April.

To explore how tropical Pacific air-sea interactions drive WNPAC formation, Zhang et al. [49] proposed a Rossby wave response mechanism to thermal anomalies during El Niño. Wang et al. [10] later identified a local ocean-atmosphere feedback mechanism. Fig 7 shows regression results of 850-hPa winds and vertical velocity (November-April) against the concurrent the ${Nino}_{3}$ index, which align with Wang et al.’s [10] framework. From late winter to early spring (January-April), the circulation response to the ${Nino}_{3}$ index strengthens (Fig 7c–f). Following eastern Pacific SST warming, a cyclonic Gill-type circulation [54] develops over the northern eastern Pacific (Fig 7c, d). The anomalous northerly winds enhance central Pacific evaporation and cool SSTs via convergence of northern waters, establishing a central Pacific cold pool [10]. This suppressed convection induces an anomalous anticyclone (WNPAC) over the northwest Pacific (Fig 7d–f). Furthermore, the weakened Walker circulation during El Niño enhances subsidence over the northwest Pacific, reinforcing the WNPAC (Fig 7c–f). Conversely, La Niña events suppress both the spring WNPAC and SEC rainfall.

### 3.4. Synergistic effect of WC and WNPAC

**Fig 8:**
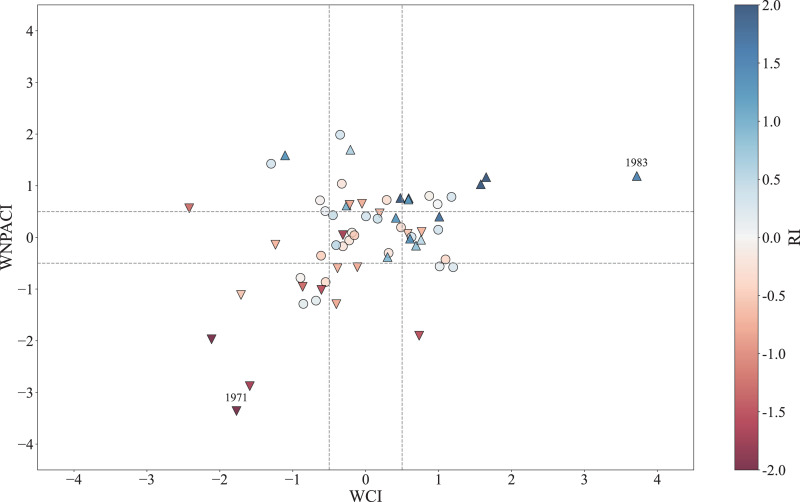
Scatter plot of standardized mid-spring WCI vs. WNPACI (1961-2022), colored by RI. Dashed lines denote ±0.5σ thresholds. Symbols indicate RI ranges: ▲ (RI > 0.5), ▼ (RI < -0.5), and ● (-0.5 < RI < 0.5). Years 1971 and 1983 are highlighted.

**Fig 9:**
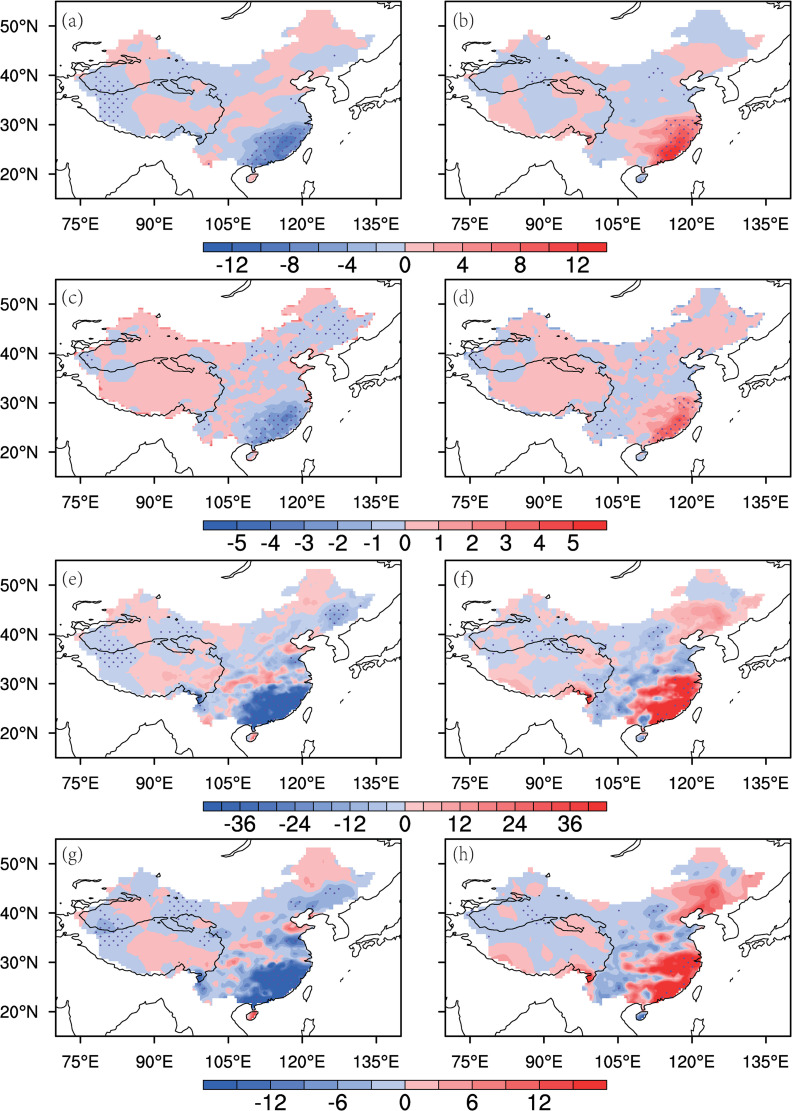
Anomaly distributions of mid-spring rainfall (a,b) and extreme precipitation indices (c-h) in China for weak (left) and strong (right) WCI-WNPACI years. (a,b) Rainfall (mm); (c,d) R20 (days); (e,f) R90TOT (mm); (g,h) Rx5day (mm). Dotted areas indicated 90% significance.

The above sections discuss the mechanisms by which the WC and WNPAC influence the interannual variability of the SEC spring rainfall. In this section, their combined effects on spring rainfall and extreme precipitation are further analyzed. Fig 8 shows the scatter plots of the spring WCI, WNPACI, and RI in the past 62 years. Using 0.5 standard deviations of the WCI and WNPACI as thresholds, the results clearly demonstrate that when both the WC and WNPAC are simultaneously strong (weak), the SEC spring rainfall tends to increase (decrease) more significantly. When they have opposite changes, the extremity of precipitation is reduced, indicating that the combined effect of the WC and WNPAC can amplify the impact of individual factors. Additionally, we select several extreme precipitation indices, such as the total number of days with precipitation exceeding 20 mm (R20), the total precipitation exceeding the 90th percentile threshold of rainy days (R90TOT), and the maximum consecutive 5-day precipitation (Rx5day), to observe the extreme precipitation anomalies during years when both WC and WNPAC are simultaneously weak or strong (Fig 9). The results show that the spring average precipitation in the SEC (Fig 9a, b) and particularly the extreme precipitation indices such as R20 (Fig 9c, d), R90TOT (Fig 9e, f), and Rx5day (Fig 9g, h), all exhibit significantly contrasting characteristics between years with strong and weak circulation indices.

**Fig 10:**
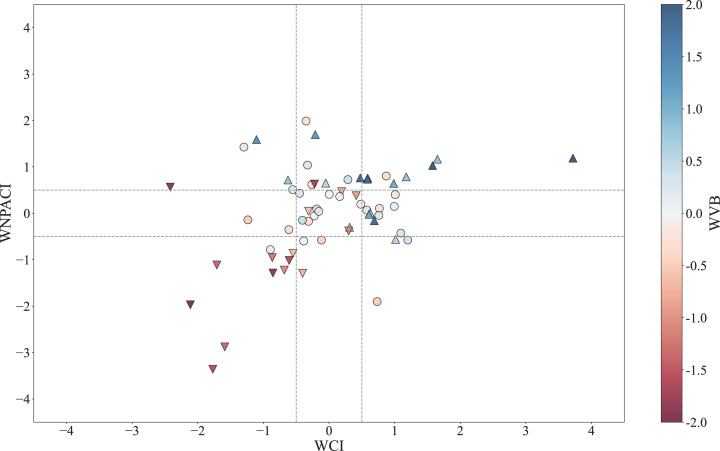
Scatter plot of standardized mid-spring WCI and WNPACI (1961–2022). Colors represent standardized WVB. Dashed lines denote ±0.5σ thresholds. Symbols: ▲ (WVB > 0.5), ▼ (WVB < -0.5), ● (-0.5 < WVB < 0.5).

Given that water vapor transport constitutes a critical determinant of SEC precipitation, particularly extreme precipitation anomalies [55], we explore the physical mechanisms underlying the synergistic effects of the WC and WNPAC on SEC precipitation and extremes through the water vapor budget (WVB) analysis. Similar to Fig 8, we present a scatter plot (Fig 10) comparing the individual and combined impacts of WC and WNPAC on WVB. The results reveal that when WNPAC is in a neutral state (- 0.5 < WNPACI < 0.5), the average WVB difference between strong and weak WC years is 0.85σ (where σ represents standard deviation of WVB). Conversely, when WC is neutral (- 0.5 < WCI < 0.5), the average WVB difference between with strong and weak WNPACI years is 0.66σ. However, when both WC and WNPAC are simultaneously strong or weak, the WVB difference increases sharply to 2.47σ, significantly exceeding the sum of their individual impacts. These results demonstrate that the synergistic interaction between WC and WNPAC amplifies water vapor transport to the SEC region during spring, thereby promoting precipitation and extreme precipitation events. This amplification likely arises from the combined dynamical effects of the WC and WNPAC, which intensify moisture convergence in the region.

**Fig 11:**
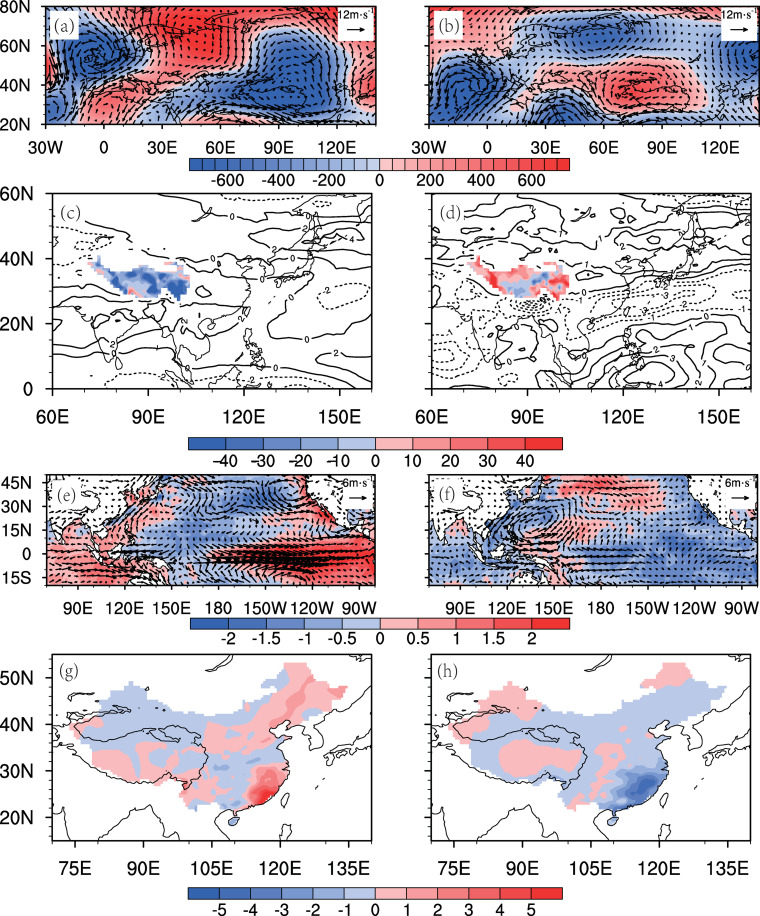
Mid-spring anomalies (relative to 1961-2022) of meteorological variables during extreme wet (1983, left) and dry (1971, right) years. (a,b) 200-hPa wind (vector, m/s) and geopotential height (shading, gpm); (c,d) 700-hPa zonal wind (contours, m/s) and SHSI over the Tibetan Plateau (>3000m, shading); (e,f) 850-hPa wind (vector, m/s) and SST (shading, K); (g,h) Precipitation (shading, mm/pentad). Dotted areas indicate 90% significance.

Table 4 lists the mid-spring SHSI, AWTI, Nino3 and RI values in years when the WC and WNPAC simultaneously strengthen or weaken. These indices consistently exhibit consistent strong or weak anomalies, indicating that the thermal effects of the Tibetan Plateau, the phase of AWT, and the strength of El Niño events are key factors influencing the SEC spring rainfall anomalies. When multiple favorable conditions align, the combined effect of the WC and WNPAC is further reinforced. By using one standard deviation as the threshold to classify these three indices into strong/weak categories, we identify two most extreme years with opposing anomaly signals, 1983 (strong) and 1971 (weak). In 1983, all three indices favored the strengthening of the WC and the WNPAC, whereas in 1971, they collectively favored weakening. Fig 11 shows the spring climate anomalies for these two years. In 1983, an anomalous low-pressure system northeast of the Tibetan Plateau and overall plateau cooling (Fig 11a, c) drove the WC reached its strongest observed intensity in recent decades (Fig 8). Concurrently, an El Niño event promoted the development of the WNPAC (Fig 11e), resulting in a significant increase in the SEC spring rainfall under the combined effect of the WC and WNPAC (Fig 11g). In contrast, 1971 exhibited opposite conditions: an anomalous high-pressure system and warming over the plateau weakened the WC (Fig 11b, d), while a La Niña event induced a cyclonic anomaly in the lower troposphere over the northwest Pacific (Fig 11f), ultimately reducing SEC rainfall (Fig 11h). These case studies demonstrate that extreme SEC spring rainfall events require concurrent anomalies in the AWT, SHS, and ENSO indices, highlighting the necessity of multi-factor synergy for extreme climatic outcomes.

**Table 4:** List of different indices in years when WC and WNPAC are both strong (weak) in spring.

	Year	SHSI	AWTI	Nino3	RI
$\left({WCI}^{+},{WNPACI}^{+}\;\right)$	1975	0.16	**2.04**	-0.59	**1.42**
	1983*	**-2.4**	**1.7**	**3.28**	**1.47**
	1986	-0.44	**1.45**	-0.55	0.01
	1987	-0.02	-0.2	**1.54**	**1.06**
	1989	-0.98	0.95	-1.17	-0.07
	1992	**-1.35**	0.99	**1.77**	**2.13**
	1993	-0.19	-0.48	0.6	0.25
	2016	1.06	-0.53	**2.56**	**2.34**
$\left({WCI}^{-},{WNPACI}^{-}\;\right)$	1963	0.64	0.38	-0.17	**-1.23**
	1971*	**1.19**	**-1.01**	**-1.77**	**-2.18**
	1999	**1.23**	0.08	-0.63	-0.03
	2000	-1.01	-0.5	**-1.24**	-0.3
	2009	0.99	**-1.66**	-0.71	**-1.5**
	2011	-0.39	**-1.73**	**-1.04**	**-2.47**
	2015	0.7	-0.41	0.33	0.15
	2018	**1.77**	-0.48	-0.97	0.14
	2021	**1.42**	0.32	-0.86	**-1.68**
	2022	**2.04**	-0.81	**-1.02**	-0.55

(+ (-) denotes strong (weak) years; The bold numbers indicate anomalies > 1σ; * marks years with exceptionally strong anomalies. Nino3: Feb-Mar mean; other indices: 15^th^–26^th^ pentad mean).

## 4. Summary and discussion

This study systematically investigates the interannual variability of the mid-spring rainfall over southeastern China (SEC) and its underlying mechanisms by analyzing 62-year observational and reanalysis data. This study advances the understanding of SEC spring rainfall by unifying previously isolated factors into a coherent framework. The findings are summarized as follows:

(1) SEC mid-spring rainfall (15^th^ -26^th^ pentad: mid-March to early May) is primarily modulated by two circulation systems: the Westerly Circulation south of the Tibetan Plateau (WC) and the Western North Pacific Anticyclone (WNPAC). These systems govern distinct water vapor transport pathways—the WC delivers mid-latitude air masses, while the WNPAC enhances tropical southwesterly influx. Their synergistic interaction amplifies precipitation extremes, explaining 52% of the interannual rainfall variance (Fig 3, Table 1).
(2) The WC is jointly influenced by the thermal forcing of the Tibetan Plateau (via surface heat source anomalies, SHS) and mid-latitude atmospheric wave trains (AWT). A cold (warm) Tibetan Plateau strengthens (weakens) the meridional temperature gradient, displacing the westerly jet southward and intensifying the WC (Figs 5 and 6). Concurrently, a positive-phase AWT generates cyclonic anomalies over the plateau, further enhancing WC and SEC rainfall (Section 3.2).
(3) The WNPAC is closely tied to tropical Pacific air-sea interactions. El Niño events trigger Rossby wave responses that establish a persistent anticyclone over the northwestern Pacific, enhancing moisture transport to the SEC. This mechanism exhibits a 1–2 month lag, offering predictive potential for spring rainfall (Fig 7, Table 3).
(4) When WC and WNPAC anomalies align (e.g., 1983 and 1971; Fig 11), their combined effect on water vapor convergence (Fig 10) and extreme precipitation indices (Fig 9) far exceeds individual contributions. Such synergy requires concurrent anomalies in SHS, AWT and ENSO, underscoring the necessity of multi-factor coordination for extreme events.

As the direct regulators of the primary water vapor transport channels, the WC and WNPAC are the direct circulation factors controlling SEC spring rainfall variations, and thus the foundational starting point of this study. However, the causes of changes in the WC and WNPAC are not limited to the wave trains discussed in this article, the thermal forcing of the Tibetan Plateau, and tropical Pacific SST anomalies. The mechanisms of these influencing factors are highly complex. For instance, the atmospheric wave train in the upper troposphere at mid-high latitudes of the Northern Hemisphere can be triggered by Arctic sea ice anomalies, preceding ENSO signals, and Atlantic SST anomalies [18,39,56]. It should be noted that the characteristics of wave trains induced by different sources of forcing may differ from one another, and not all external forcings are directly related to the SEC spring rainfall. The surface heat source anomaly of the Tibetan Plateau may be linked to winter-to-spring snow cover changes on the plateau or the Eurasian continent [19,20]. The thermal forcing of the Tibetan Plateau directly impacts on the surrounding atmospheric circulation, while large-scale thermal anomalies over Eurasia may also cause westerly circulation anomalies [57,58].

Regarding WNPAC, ENSO and Indian Ocean SSTs are likely important influencing factors, but there are also significant interactions between ENSO and Indian Ocean SSTs. For example, following ENSO events, the Indian Ocean often exhibits the Indian Ocean Basin Mode in spring [59,60]. As for ENSO, a key factor affecting the WNPAC, its formation mechanism is also particularly complex. Previous studies have shown that there is still considerable uncertainty in the ENSO variability by different model estimates [61]. Furthermore, winter Arctic sea ice concentrations, SST anomalies in the Indian or Atlantic oceans may have also influence the development of ENSO [62,63]. Therefore, in future research, it is necessary to further distinguish these external forcing sources and consider the potential synergistic effects of different forcings on SEC spring precipitation.

This study focuses on the interannual variability of the SEC spring rainfall rather than its long-term trend. Previous studies have identified a significant decreasing trend in early spring (February-April) rainfall in the SEC, which may be linked to the influence of anomalous high-pressure systems over the North Pacific and East Asia [64]. Other studies suggest that the weakening of the westerly circulation in recent decades has played a key role [21]. However, the long-term trend of the mid-spring SEC precipitation (1961–2022) discussed in this study is not significant (p > 0.1), although some decadal variability is evident (Fig 3a). The 62-year period analyzed in this study can be divided roughly into five stages: 1962–1972, 1973–1996, 1998–2011, 2011–2017, and 2019–2022, with precipitation anomalies showing a pattern of “-, +, -, +, -”. Additionally, it is also noted that the long-term trend of mid-spring westerly circulation shows a significant weakening (p < 0.05) (Fig 3b), consistent with the findings of Zeng et al. [21]. This implies that, compared to early spring, the westerly circulation in mid-spring is not a determining factor for the long-term trend of SEC rainfall.

## References

[1] Tian SF, Yasunari T. Climatological Aspects and Mechanism of Spring Persistent Rains over Central China. Journal of the Meteorological Society of Japan. 1998;76(1):57–71. doi: 10.2151/jmsj1965.76.1_57

[2] Feng J, Li J. Influence of El Niño Modoki on spring rainfall over south China. J Geophys Res. 2011;116(D13). doi: 10.1029/2010jd015160

[3] Pan WJ, Mao JY, Wu GX. Characteristics and Mechanism of the 10–20-Day Oscillation of Spring Rainfall over Southern China. Journal of Climate. 2013;26:5072–87. doi: 10.1175/JCLI-D-12-00618.1

[4] Zhang SW, Wang H, Jiang H, Ma WT. Studies of the seasonal prediction of heavy late spring rainfall over southeastern China. Climate Dynamics. 2021;57:1919–31. doi: 10.1007/s00382-021-05786-w

[5] Li PX, Zhou TJ, Chen XL. Water vapor transport for spring persistent rains over southeastern China based on five reanalysis datasets. Climate Dynamics. 2018;51:4243–57. doi: 10.1007/s00382-017-3680-3

[6] Park CK, Park DSR, Ho CH, Park TW, Kim J, Jeong S, et al. A Dipole Mode of Spring Precipitation between Southern China and Southeast Asia Associated with the Eastern and Central Pacific Types of ENSO. Journal of Climate. 2020;33:10097–111. doi: 10.1175/JCLI-D-19-0625.1

[7] Guo R, Pan W, Ke M, Wei W, Wang Z. Diversity on the interannual variations of spring monthly precipitation in southern China and the associated tropical sea surface temperature Anomalies. Journal of Tropical Meteorology. 2023;29:337–46. doi: 10.3724/j.1006-8775.2023.025

[8] Zhong W, Wu Y, Yang S, Ma T, Cai Q, Liu Q. Heavy Southern China Spring Rainfall Promoted by Multi‐Year El Niño Events. Geophysical Research Letters. 2023;50(7). doi: 10.1029/2022gl102346

[9] Li G, Feng L, Zhuang W, Liu F, Zhang R, Sui C. Differences in spring precipitation over southern China associated with multiyear La Niña events. Acta Oceanol Sin. 2024;43(2):1–10. doi: 10.1007/s13131-023-2147-0

[10] Wang B, Wu RG, Fu XH. Pacific–East Asian Teleconnection: How Does ENSO Affect East Asian Climate? Journal of Climate. 2000;13:1517–36. doi: 10.1175/1520-0442(2000)013≤1517:PEATHD≥2.0.CO;2

[11] Wu RG, Hu ZZ, Kirtman BP. Evolution of ENSO-Related Rainfall Anomalies in East Asia. Journal of Climate. 2003;16(22):3742–58. doi: 10.1175/1520-0442(2003)016≤3742:EOERAI≥2.0.CO;2

[12] Wu XF, Mao JY. Interdecadal modulation of ENSO-related spring rainfall over South China by the Pacific Decadal Oscillation. Climate Dynamics. 2016;47:3203–20. doi: 10.1007/s00382-016-3021-y

[13] Wu XF, Mao JY. Spatial and interannual variations of spring rainfall over eastern China in association with PDO–ENSO events. Theoretical and Applied Climatology. 2017;134:935–53. doi: 10.1007/s00704-017-2323-2

[14] Feng JQ, Yu LJ, Hu DX. Influence of Indian Ocean subtropical dipole on spring rainfall over China. International Journal of Climatology. 2014;34:954–63. doi: 10.1002/joc.3732

[15] Tang W, Fu Y, Wang X, Lu Y, Xu M, Xie W, et al. Decreasing spring persistent rainfall over the Yangtze‐Huai River Valley of China during 1960–2019 and its possible causes. Intl Journal of Climatology. 2021;42(7):3809–19. doi: 10.1002/joc.7446

[16] Jia X, Zhang C, Wu R, Qian Q. Changes in the Relationship between Spring Precipitation in Southern China and Tropical Pacific–South Indian Ocean SST. Journal of Climate. 2021;34(15):6267–79. doi: 10.1175/jcli-d-20-0817.1

[17] Hao SB, Li JD, Mao JY, Liu YM, Wu GX. Interannual variability of spring rainfall over South China in association with the North Pacific Oscillation and North Atlantic Oscillation as revealed by reanalysis data and CMIP6 simulations. Climate Dynamics. 2024;62:7535–57. doi: 10.1007/s00382-024-07293-0

[18] Wu ZW, Li XX, Li YJ, Li Y. Potential Influence of Arctic Sea Ice to the Interannual Variations of East Asian Spring Precipitation. Journal of Climate. 2016;29(8):2797–813. doi: 10.1175/JCLI-D-15-0128.1

[19] Jia X, Zhang C, Wu R, Qian Q. Influence of Tibetan Plateau autumn snow cover on interannual variations in spring precipitation over southern China. Climate Dynamics. 2021;56:767–82. doi: 10.1007/s00382-020-05497-8

[20] Zuo ZY, Zhang RH, Wu BY, Rong XY. Decadal variability in springtime snow over Eurasia: Relation with circulation and possible influence on springtime rainfall over China. International Journal of Climatology. 2011;32:1336–45. doi: 10.1002/joc.2355

[21] Zeng ZY, Yang S, Wang Z, Luo H, Deng K. Weakened Subtropical Westerlies and Their Deflection by the Tibetan Plateau Contribute to Drying Southeastern China in Early Spring. Geophysical Research Letters. 2024;51:e2024GL109795. doi: 10.1029/2024GL109795

[22] Wan RJ, Wu GX. Mechanism of the Spring Persistent Rains over southeastern China. Science in China Series D Earth Sciences. 2007;50:130–44. doi: 10.1007/s11430-007-2069-2

[23] Wan RJ, Zhao B, Wu GX. New evidences on the climatic causes of the formation of the spring persistent rains over southeastern China. Advances in Atmospheric Sciences. 2009;26:1081–7. doi: 10.1007/s00376-009-7202-z

[24] Wu J, Gao X. A gridded daily observation dataset over China region and comparison with the other datasets. Chin J Geophys. 2013;56(4):1102–11.

[25] Liu Z, Di Z, Qin P, Zhang S, Ma Q. Evaluation of Six Satellite Precipitation Products over the Chinese Mainland. Remote Sensing. 2022;14:6277. doi: 10.3390/rs14246277

[26] Jiang S, Han T, Zhou B, Zhang Q, Hao X, Li H. Characteristics of clustered heavy precipitation events at Northeast China and associated atmospheric circulations. Climate Dynamics. 2023;61:5921–33. doi: 10.1007/s00382-023-06944-y

[27] Zhu C, Yue Q, Huang J. Projections of Mean and Extreme Precipitation Using the CMIP6 Model: A Study of the Yangtze River Basin in China. Water. 2023;15:3043. doi: 10.3390/w15173043

[28] Hersbach H, Bell B, Berrisford P, Biavati G, Horányi A, Muñoz SJ, et al. ERA5 hourly data on pressure levels from 1940 to present. Copernicus Climate Change Service (C3S) Climate Data Store (CDS). 2023. doi: 10.24381/cds.bd0915c6

[29] Rayner NA, Parker DE, Horton EB, Folland CK, Alexander LV, Rowell DP, Kent EC, Kaplan A. Global analyses of sea surface temperature, sea ice, and night marine air temperature since the late nineteenth century. Journal of Geophysical Research. 2003;108:2002JD002670. doi: 10.1029/2002JD002670

[30] Capotondi A, Sardeshmukh DP. Optimal precursors of different types of ENSO events. Geophysical Research Letters. 2015;42:9952–60. doi: 10.1002/2015GL066171

[31] Takaya K, Nakamura H. A Formulation of a Phase-Independent Wave-Activity Flux for Stationary and Migratory Quasigeostrophic Eddies on a Zonally Varying Basic Flow. J Atmos Sci. 2001;58(6):608–27. doi: 10.1175/1520-0469(2001)058<0608:afoapi>2.0.co;2

[32] Ye DZ, Gao YX. Meteorology of the Tibetan Plateau (in Chinese). Science Publication Agency; 1979. p. 1–278.

[33] Rasmusson EM. Atmospheric water vapor transport and the water balance of North America. II. Large-scale water balance investigations. Monthly Weather Review. 1968;96:720–734. doi: 10.1175/1520-0493(1968)096≤0720:AWVTAT≥2.0.CO;2

[34] Ding YH, Liu JJ, Ying S, Liu YJ, He JH, Song YF. A study of the synoptic-climatology of the Meiyu system in East Asia. Chinese Journal of Atmospheric Sciences. 2007;31:1082–01. doi: 10.3878/j.issn.1006-9895.2007.06.05

[35] Ding YH, Liang P, Liu YJ, Zhang YC. Multiscale variability of Meiyu and its prediction: A new review. Journal of Geophysical Research: Atmospheres. 2020;125:e2019JD031496. doi: 10.1029/2019JD031496

[36] Li Q, Zhang RH. Seasonal variation of climatological bypassing flows around the Tibetan Plateau. Advances in Atmospheric Sciences. 2012;29:1100–10. doi: 10.1007/s00376-012-1154-4

[37] Bao Y, You Q. How do westerly jet streams regulate the winter snow depth over the Tibetan Plateau? Clim Dyn. 2019;53(1–2):353–70. doi: 10.1007/s00382-018-4589-1

[38] Chen S, Wu R, Chen W, Hu K, Yu B. Structure and dynamics of a springtime atmospheric wave train over the North Atlantic and Eurasia. Clim Dyn. 2020;54(11–12):5111–26. doi: 10.1007/s00382-020-05274-7

[39] Zhang RH, Min QY, Su JZ. Impact of El Niño on atmospheric circulations over East Asia and rainfall in China: Role of the anomalous western North Pacific anticyclone. Science China Earth Sciences. 2017;60:1124–32. doi: 10.1007/s11430-016-9026-x

[40] Kuang X, Zhang Y. Seasonal variation of the East Asian Subtropical Westerly Jet and its association with the heating field over East Asia. Advances in Atmospheric Sciences. 2005;22:831–40. doi: 10.1007/BF02918683

[41] Chen SF, Wu RG, Liu Y. Dominant Modes of Interannual Variability in Eurasian Surface Air Temperature during Boreal Spring. Journal of Climate. 2016;29(3):1109–25. doi: 10.1175/JCLI-D-15-0524.1

[42] Liu YM, Bao Q, Duan AM, Qian ZA, Wu GX. Recent progress in the impact of the Tibetan Plateau on climate in China. Advances in Atmospheric Sciences. 2007;24:1060–76. doi: 10.1007/s00376-007-1060-3

[43] Xie XN, Duan AM, Shi ZG, Li XZ, Sun H, Liu XD, et al. Modulation of springtime surface sensible heating over the Tibetan Plateau on the interannual variability of East Asian dust cycle. Atmospheric Chemistry and Physics. 2020;20:11143–59. doi: 10.5194/acp-20-11143-2020

[44] Shang K, Liu X, Dong B. Climatology and physical mechanisms of the tropospheric warm cores over the Tibetan Plateau and its vicinity. Clim Dyn. 2021;57(3–4):953–74. doi: 10.1007/s00382-021-05749-1

[45] Lan M, Zhang Y. Relationship between the East Asian subtropical westerly jet and summer rainfall anomaly in Northeast China. J Meteorol Sci. 2011;31:258–65. doi: 10.3969/j.issn.1009-0827.2011.03.003

[46] Li XZ, Liu XD. Numerical simulation of Tibetan Plateau heating anomaly influence on westerly jet in spring. Journal of Earth System Science. 2015;124(8):1599–607. doi: 10.1007/s12040-015-0630-5

[47] Li X, Liu X, Pan Z, Shi Z, Xie X, Guo Q. A transient simulation of precession-scale spring dust activity over northern China and its relation to mid-latitude atmospheric circulation. Palaeogeography, Palaeoclimatology, Palaeoecology. 2020;542:109585. doi: 10.1016/j.palaeo.2020.109585

[48] Chen SF, Wu RG. Interdecadal Changes in the Relationship between Interannual Variations of Spring North Atlantic SST and Eurasian Surface Air Temperature. Journal of Climate. 2017;30(10):3771–87. doi: 10.1175/JCLI-D-16-0477.1

[49] Zhang RH, Sumi A, Kimoto M. Impact of El Niño on the East Asian Monsoon: A Diagnostic Study of the ’86/87 and ’91/92 Events. Journal of the Meteorological Society of Japan. 1996;74:49–62. doi: 10.2151/jmsj1965.74.1_49

[50] Xie M, Wang C. Decadal Variability of the Anticyclone in the Western North Pacific. Journal of Climate. 2020;33(20):9031–43. doi: 10.1175/jcli-d-20-0008.1

[51] Feng J, Chen W. Roles of the North Indian Ocean SST and Tropical North Atlantic SST in the Latitudinal Extension of the Anomalous Western North Pacific Anticyclone during the El Niño Decaying Summer. Journal of Climate. 2021;34(21):8503–17. doi: 10.1175/JCLI-D-20-0802.1

[52] Feng J, Chen W. Respective and Combined Impacts of North Indian Ocean and Tropical North Atlantic SST Anomalies on the Subseasonal Evolution of Anomalous Western North Pacific Anticyclones. Journal of Climate. 2022;35(17):5623–36. doi: 10.1175/jcli-d-21-0799.1

[53] Zhang RH, Sumi A. Moisture Circulation over East Asia during El Nino Episode in Northern Winter, Spring and Autumn. Journal of the Meteorological Society of Japan. 2002;80:213–27. doi: 10.2151/jmsj.80.213

[54] Gill AE. Some simple solutions for heat‐induced tropical circulation. Quart J Royal Meteoro Soc. 1980;106(449):447–62. doi: 10.1002/qj.49710644905

[55] Cao DR, Francis Tam CY, Xu K. Simulating springtime extreme rainfall over Southern East Asia: unveiling the importance of synoptic-scale activities. Climate Dynamics. 2024;62:9073–96. doi: 10.1007/s00382-024-07379-9

[56] Cai QY, Chen W, Chen SF, Ma TJ, An XD, Li ZB. The Strengthened Linkage between ENSO and the Eurasian Pattern since the Late 1980s. Journal of Climate. 2024;37(24):6491–502. doi: 10.1175/JCLI-D-23-0402.1

[57] Yang J, Chen H, Song Y, Zhu S, Zhou B, Zhang J. Atmospheric Circumglobal Teleconnection Triggered by Spring Land Thermal Anomalies Over West Asia and Its Possible Impacts on Early Summer Climate Over Northern China. Journal of Climate. 2021:1–80. doi: 10.1175/jcli-d-20-0911.1

[58] Song YD, Chen HS, Yang JQ. The dominant modes of boreal spring land surface temperature over Western Eurasia and their possible linkages with large-scale atmospheric teleconnection patterns. Journal of Geophysical Research: Atmospheres. 2022;127(4):e2021JD035720. doi: 10.1029/2021JD035720

[59] Xie SP, Kosaka Y, Du Y, Hu K, Chowdary JS, Huang G. Indo-western Pacific Ocean capacitor and coherent climate anomalies in post-ENSO summer: a review. Advances in Atmospheric Sciences. 2016;33:411–32. doi: 10.1007/s00376-015-5192-6

[60] Marathe S, Terray P, Karumuri A. Tropical Indian Ocean and ENSO relationships in a changed climate. Climate Dynamics. 2021;56:3255–76. doi: 10.1007/s00382-021-05641-y

[61] Beobide-Arsuaga G, Bayr T, Reintges A, Latif M. Uncertainty of ENSO-amplitude projections in CMIP5 and CMIP6 models. Climate Dynamics. 2021;56:3875–88. doi: 10.1007/s00382-021-05673-4

[62] Terray P, Masson S, Prodhomme C, Roxy MK, Sooraj KP. Impacts of Indian and Atlantic oceans on ENSO in a comprehensive modeling framework. Clim Dyn. 2015;46(7–8):2507–33. doi: 10.1007/s00382-015-2715-x

[63] Chen SF, Wu RG, Chen W, Yu B. Influence of winter Arctic sea ice concentration change on the El Niño–Southern Oscillation in the following winter. Climate Dynamics. 2020;54:741–57. doi: 10.1007/s00382-019-05027-1

[64] Li YN, Zhou W, Yang S, Zhang RH, Cheung HN, Zhang Y. Recent Early-Spring Drying Trend over Southern China Associated with Changes in the Zonal Thermal Contrast over the Pacific. Journal of Climate. 2022;35(19):6487–9. doi: 10.1175/JCLI-D-21-0891.1

